# Data science as a language: challenges for computer science—a position paper

**DOI:** 10.1007/s41060-018-0103-4

**Published:** 2018-02-28

**Authors:** Arno Siebes

**Affiliations:** Department of Information and Computing Sciences, Universiteit Utrecht, Utrecht, The Netherlands

**Keywords:** Data mining, Data science, Inductive inference

## Abstract

In this paper, I posit that from a research point of view, Data Science is a language. More precisely Data Science is doing Science using computer science as a language for datafied sciences; much as mathematics is the language of, e.g., physics. From this viewpoint, three (classes) of challenges for computer science are identified; complementing the challenges the closely related Big Data problem already poses to computer science. I discuss the challenges with references to, in my opinion, related, interesting directions in computer science research; note, I claim neither that these directions are the most appropriate to solve the challenges nor that the cited references represent the best work in their field, they are inspirational to me. So, what are these challenges? Firstly, if computer science is to be a language, what should that language look like? While our traditional specifications such as pseudocode are an excellent way to convey what has been done, they fail for more mathematics like reasoning about computations. Secondly, if computer science is to function as a foundation of other, datafied, sciences, its own foundations should be in order. While we have excellent foundations for supervised learning—e.g., by having loss functions to optimize and, more general, by PAC learning (Valiant in Commun ACM 27(11):1134–1142, 1984)—this is far less true for unsupervised learning. Kolmogorov complexity—or, more general, Algorithmic Information Theory—provides a solid base (Li and Vitányi in An introduction to Kolmogorov complexity and its applications, Springer, Berlin, 1993). It provides an objective criterion to choose between competing hypotheses, but it lacks, e.g., an objective measure of the uncertainty of a discovery that datafied sciences need. Thirdly, datafied sciences come with new conceptual challenges. Data-driven scientists come up with data analysis questions that sometimes do and sometimes don’t, fit our conceptual toolkit. Clearly, computer science does not suffer from a lack of interesting, deep, research problems. However, the challenges posed by data science point to a large reservoir of untapped problems. Interesting, stimulating problems, not in the least because they are posed by our colleagues in datafied sciences. It is an exciting time to be a computer scientist.

## Introduction

The most important observation one can make regarding Data Science is that its advent signals the end of the era in which Computer Science was under the purview of computer scientists. Started by, among others, mathematicians, physicists, and engineers, computer science evolved into an independent research area [60]. An area with its own traditions and culture determining what is considered good research, what the important problems are, and with its own publishing standards.

However, now, due to the digitization—or perhaps more apt, *datafication*—of all of academia, computer science is quickly becoming an integral part of all areas of academic endeavor. Storing, manipulating and analyzing vast amounts of data of a bewildering variety of types are becoming the core of many new approaches to science, any kind of science.

Bioinformatics [36, 38] is probably the first, and arguably the biggest, example of the datafication of a science, but other sciences are following quickly. From the Social Sciences (computational social science [15]), to the Humanities (digital humanities [13]) to Education (learning analytics [55]) to Astronomy (sky surveys [71]). All of these areas have their own problems, invent their own solutions and have their own view on what the important problems are, what marks a good solution, and have their own publishing standards and culture. Some of these problems, and their solutions, can be considered as computer science while others can not. But, when it is computer science, it is computer science performed and published outside the computer science community.

The umbrella term catching all these disparate research areas is Data Science. That is, to me, data science is the union of datafied research in all of academia. The question I discuss in this position paper is what impact the advent of data science has on computer science. What challenges does data science pose to computer science? Note that I only discuss challenges to computer science research, not to education. This is not to be construed as that I am against Master or Bachelor programs offering a solid base in the computational aspects of Data Science. Far from it, like (almost) everyone else, I’m enthusiastically involved in such initiatives. It just means that I think that the research challenges posed are even more exciting.

Clearly, data science is still very new; hence, this is just a preliminary list of some challenges, far from an exhaustive survey. Many challenges, of which we have no inkling at the moment, will appear while data science evolves into its own independent research field. Moreover, I’ll limit myself mostly to that part of computer science I am most familiar with, viz., machine learning and data mining. I do hope, however, that the challenges I discuss illustrate the breadth and the depth of the challenges for all of computer science. Even more I hope they illustrate that the advent of data science is good for computer science; if possible, it makes it even more fun to be a computer scientist.

I will restrict myself to the academic context. This is *not* to be read as stating that data science is not relevant outside academia. Far from it, data science is arguably far more important outside academia than inside. The reason to restrict myself to academia is that this context makes it possible to point out challenges that are more difficult to discern in the torrent of non-academic applications. I am sure that the points I make and the challenges I identify are equally valid in the wider context.

Note that the restriction to the academic setting has as a consequence that we restrict ourselves mostly to the data-driven science aspects of data science. Clearly, there is more to data science than just data-driven science. The science *of* data is an example of the wider notion and one that points to interesting challenges as well. A discussion of those challenges is, however, beyond the scope of this paper.

The reader may already have noted I (mis)use the term science to stand for all areas of academic endeavor, very much like the word Wissenschaften in German or Wetenschappen in Dutch. While the term Science is generally used in the more restricted sense of natural sciences, this broader understanding is cromulent according to the Oxford English Dictionary. Moreover, using this broad understanding of the term Science makes writing—and hopefully reading—this position paper a lot easier.

Finally, in an area like machine learning, it is hard to make a distinction between computer science and statistics. The gap between two cultures Leo Breiman discerned [12] seems to have all but gone, because of the ever growing dependence on computational methods.^1^ A process that is, perhaps, best illustrated in the chronological presentation of methods in [23]. So, while I use the terms computer science and computer scientist, the whole discussion is equally valid for statistics and statisticians; in fact, equally valid for anyone working on the foundations of data science. Restricting my self to the term computer science—rather than writing “computer science and statistics” everywhere—again makes the writing (and hopefully the reading) of this paper easier. I hope that this choice is not a major put-off to statisticians.

## Data Science

To formulate challenges that data science poses to computer science, it is necessary to discuss data science itself. What is this data science?

Historically, data science has been posed as an alternative name for computer science by Peter Naur and, later, by C.-F. Jeff Wu and others, as an alternative name for statistics. Now, it is most often used as a name for the field that arises out of the datafication of all the sciences.

The most important implication of that observation is that data science is *not* computer science. The close second is that data science is *not* an interdisciplinary—or multidisciplinary—affair. To start with the former, a historian is out to answer historical questions, not to answer computer science questions. She may need to solve CS problems to answer her historical questions, but the interest is on the latter. That is, both the problems and their proposed solutions are evaluated from the point of view of a historian.

This doesn’t preclude that an area like history informatics—which is solely concerned with developing solutions for historians—may arise in the not to distant future. It does preclude that area will be just another computer science area. A journal like Bioinformatics is really different from a regular computer science journal, relegating crucial—to computer scientists(!)—information to the Supporting Information.^2^ Moreover, contributions are not primarily evaluated on their computer science merits, learning analytics tools that do not align with current pedagogical theories will not be seen as valid, however elegant the computer science aspects are.

This may be seen as an argument to see data science as an interdisciplinary field. But it isn’t. True, each separate field—like bioinformatics or history informatics—may be seen as an interdisciplinary field, albeit with its own culture and standards as noted above. A computer scientist willing to learn enough of the host science to understand the problems and what makes a valid solution can probably make important contributions. But solutions that are valid in one corner of data science—say, bioinformatics—is not necessary a valid solution in another corner, say history informatics.

This is also the reason why data science is not multidisciplinary. Biologists with data problems do not necessarily always have much in common with historians with data problems. Some problems and/or their solutions will be common; others will be area specific.

It may be nice to know what it isn’t, but it is more useful to know what it is. So, *what is data science?* That is easy:

Data Science is a language

A computer science kind of language, its vocabulary contains words like *algorithms, data structures, queries,* and so on. However, the sentences uttered in that language are *not* necessarily computer science. Rather,

Data Science is a language in which scientists – often from disciplines other than computer science – can talk about problems and their solutions in their discipline that involve the storage, manipulation, or analysis of (large amounts of) data.

There will be many dialects of Data Science, dialects that allow a scientist to formulate problems in her own science precisely. But all these dialects will contain a common core, viz., algorithmic thinking and algorithms. A core that allows scientists to talk about the computational problems and solutions involved in solving their science problem. A core that is Computer Science. That is,

Data Science is Computer Science as a language.

### What is computer science as a language?

To explain what we mean with “computer science as a language”, we start with an analogy.

Without a thorough grounding in mathematics, it is impossible to read a basic law of physics, like Maxwell’s

$$\begin{aligned} \nabla \times E = \frac{-\partial B}{\partial t} \end{aligned}$$

In fact, without proficiency in mathematics, it is impossible to do physics. For example, to derive that a body of mass *M* bends a ray of light at distance *r* by

$$\begin{aligned} \theta = \frac{4 GM }{c^2r} \end{aligned}$$

from Einstein’s general relativity law:

$$\begin{aligned} G_{\mu \nu } + \varLambda g_{\mu \nu } = \frac{8\pi G}{c^4} T_{\mu \nu } \end{aligned}$$

one needs to be rather adept in manipulating expressions according to the “laws” of mathematics.

Physics, or more poetically The Book of Nature, is written in mathematics. Mathematics is the language of physics. As Plato allegedly wrote above the door to his academy: $\mathrm{A} \Gamma \mathrm{E} \Omega \mathrm{M} \mathrm{E} \mathrm{T} \mathrm{P} \mathrm{H} \mathrm{T} \mathrm{O} \Sigma \mathrm{M} \mathrm{H} \Delta \mathrm{E} \mathrm{I} \Sigma \mathrm{E} \mathrm{I} \Sigma \mathrm{I} \mathrm{T} \Omega$.

Note that deriving $\theta$ from Einstein’s law is doing physics, not doing mathematics. The derivation adds to the body of knowledge of physics and not to the body of knowledge of mathematics.^3^

Just as physicists use mathematics as a language, datafied scientists will use computer science as a language. Without a thorough grounding in computer science, it will become impossible to read and do science in the datafied sciences. And just as physicists don’t do mathematics, datafied scientists don’t do computer science, they do research in their own field.

But, note, that they do their research in their own field in a fundamentally new way. Data-driven research in *X* is different from the traditional way of doing research in *X*. Not only because of the use of, e.g., computers and databases, but also conceptually: the data-driven approach supports questions that simply couldn’t be answered before; see Sect. 3.2 for an example.

It is because this second aspect that Data Science deserves its own name. If data science would “just” be about using computer science tools to solve traditional problems in, say, History, the term computer science would probably be sufficient; after all physicists use mathematics, no new name. The term Data Science emphasizes that the datafication of sciences allow the study of new types of problems for which the solutions are grounded in computer science

Note that to some extent this has already started to happen with the advent of Computational Science [46] in which the complexities of nature are studied using computational models and simulations. Data science, however, will have a much further reach in all sciences and uses a larger part of computer science as well.

Note that the relation between computer science and the other sciences may run deeper than the relation between mathematics and physics. In recent years, *information* is more and more identified as a foundational concept in many other sciences. For example, in [25], the Nobel-prize winner Manfred Eigen lucidly builds bridges between physics and biology based on a new theory of information; some of the themes from this book are further expanded upon in [26]. While most of those contributions are conceptual,  Verlinde [67] introduces a concrete model in which gravity emerges from changes in the information associated with the positions of material bodies, showing, e.g., that this emergent force satisfies Newton’s laws.

While one could debate whether or not these examples are data science, our final example most definitely does. In [44], the author proposes a framework for theoretical physics grounded in Algorithmic Information Theory, one of the foundational theories for Data Science.^4^ Thus putting algorithmic reasoning at the very heart of physics.

### What does this language look like?

The discussion in the previous subsection was mostly conceptual. Languages, however, have syntax and semantics. What will those be for computer science as a language? How will it look like? The short answer is: I do not know. In This subsection, I discuss two aspects that I think will be important for this new language. The first is about syntax, the second about semantics.

If one would extend the analogy with mathematics further, one would expect a symbolic language and mostly modestly sized expressions in that language. For example, although mathematicians have, e.g., named and studied a bewildering variety of (special) functions—see the iconic handbook of functions by Abramowitz and Stegun [1] or its modern day counterpart (also) produced by NIST [45]^5^—one can read vast parts of the mathematical literature knowing only a limited set of, e.g., symbols and functions.

Moreover, the symbolic expressions tend to be concise; the page long Lagrangian in Appendix E of [66] is in my experience an exception. Derivations—i.e., reasoning—may be spread over multiple pages, but the individual expressions in a derivation are usually modestly sized. The fact that the reasoner has to keep track of all bits and pieces is undoubtedly one of the major reasons for the conciseness; abstraction is king.

Unfortunately, symbolic or formal languages in computer science tend to be less concise. While algorithms are usually described in some (semi-formal) pseudocode, they easily run into a page or more. Data scientists using existing algorithms clearly do not have to reproduce the complete pseudocode, rather, they “just” have to give the algorithms used as well as their parameter settings and data sets; very much in the way that computer scientists themselves describe their experiments with their newly developed algorithms.

Note that the fact that there tend to be hundreds of algorithms and variants for the same algorithmic task doesn’t make this approach much easier to read (or write). One could aim to standardize naming, leading to expressions likeGiven the multitude of broadly ranging details of a complete Knowledge Discovery process, this may again lead to rather large and hard to parse expressions. An alternative is the visual programming—work flow style—interface of data mining tools such as KNIME [7]. Such annotated graphs are, perhaps, a better language—leading to more comprehensible expressions—for computer science expressions in data science than the script-like expressions above.

Taking the analogy with mathematics further, physicists do not only use mathematical expressions, they also reason with them. Reasoning with the models a machine learning algorithm delivers is very much part of what computer scientists already do; model inference is part and parcel of the field. Reasoning with the algorithms themselves—or, more general, the whole KDD process—is, however, far less developed.

Clearly, neither the use of parametrized tasks as suggested above, nor the work flows of KNIME allow one to reason with algorithms in the way physicists reason with mathematical expressions. The development of that aspect of computer science as a language is one of the challenges data science poses to computer science.

Perhaps the research area in computer science closest to this challenge is that of *program transformation* [9]. Here, the goal is to derive an efficient implementation from an obviously correct specification. This goal is similar to the rewriting of queries in relational algebra for query optimization [29], but with a much larger collection of operators and laws. To illustrate, using operators like $\oplus$, satisfying laws like

$$\begin{aligned}&\oplus /[a] = a \\&\oplus /(x ++ y) = (\oplus /x) \oplus (\oplus /y) \end{aligned}$$

one performs derivations like

$$\begin{aligned} x \otimes y&= (\otimes / f \varvec{\cdot } x') \otimes (\otimes / f \varvec{\cdot } y') \\&\vdots \\&= ((\oplus /) \varvec{\cdot } \alpha x') ++ (\oplus /) \varvec{\cdot } (x +\!\!\!+) \varvec{\cdot } \alpha y' \end{aligned}$$

to prove that

$$\begin{aligned} x \otimes y = x ++((\text {last } x) \oplus ) \varvec{\cdot } y \end{aligned}$$

An expression that states the computational equivalence between a quadratic specification (on the left) to a linear implementation (on the right); see [9] for full details.

The question is: what would be the right operators—at what level of abstraction—and what laws do they satisfy, to allow for a similar type of reasoning about a data analysis task?

## Challenges for computer science

The observation “Data Science is not Computer Science” does not mean that there is no computer science in data science. Far from it. In the Introduction I already noted that to me data science is the union of datafied research in all of academia. One of these research areas is, of course, computer science. In fact, as I will argue in this section, data science poses new as well as already existing challenges to computer science. Challenges that must be met to let computer science truly become a language for data science.

First of all, again in the Introduction, we already noted that storing, manipulating and analyzing vast amounts of data of a bewildering variety of types is becoming the core of many new approaches to science, any kind of science. That is, the problems colloquially known as Big Data are an integral part of the challenges posed by data science.

Despite that Big Data is a term coined by consultancy firms rather than by the research community as well as serious concerns about it raised by researchers—see, e.g., [11]—the term is an umbrella for a set of serious problems. Each of the commonly used three V’s to describe the area, points to a set of related problems.

The first V stands for Volume. The data is too big to handle. Scalability of algorithms is an obvious related research area here, with topics such as sublinear algorithms [18] and property testing [30]. Another big topic is, of course, sampling—to which we return below.

The second V stands for Velocity, the high volumes of data stream in at high rate. Either you look at it now or the opportunity is gone forever. This means again that scalability is an important aspect, but so are, e.g., anytime algorithms [72] and real time computing [14].

The final V stands for Variety. There are many algorithms to mine many different types of data, such as transaction data [4], text data [28], time series data [40], sequence data (sequences of, again, a wide variety of types) [21], graph data [16], network data [6, 22] and multi relational data [20]. There are, however, few if any algorithms that can deal with multiple data streams that combine such different data types.

While combining techniques for different data types is in principle easy, it is far less easy to guarantee good results. It is, e.g., well known in regression analysis that one should scale the independent variables so that they all have a similar range. If the variables are not scaled, the variable with the largest range will have undue influence on the chosen model [59]. Whether one should “scale”, what it would actually mean—what is the scale of text?—and how it should be done over disparate data sources are still very much open problems.

And, then, we have not yet talked about the problem of Veracity [8]; sometimes known as the fourth V. Data is often uncertain or imprecise, due to noise and other factors. Keeping track in a multi-source setting, of what part of the data is derived from what (uncertain) source is the non-trivial problem known as provenance [31]. Another problem is that traditional methods are usually well-equipped to handle a single noisy data source, but combining different data sources with different levels of veracity is again still a very much open problem.

It is not just Big Data that makes it challenging for data-driven scientists to use our methods. We already briefly discussed another one in the context of the language, viz., the large variety of algorithms for the same data mining problem—such as classification. This is a challenge for those who want to use machine learning algorithms to solve their own problems. Which one should they use and with what parameter settings? This is also still very much an open question for the computer scientists working in the area.

One approach to solve it is OpenML.^6^ OpenML offers various services to share and find data sets, to download or create scientific tasks, to share and find implementations (called flows), and to share and organize results [63]. By having a comparison of many implementations over many different data sets with many different parameter settings, a user should get an idea of what might be the good approach for her.

Another approach is to let the computer automatically explore the vast search space of possibilities to find the best fit for the data at hand. One project exploring this approach is the Automatic Statistician project by the University of Cambridge and MIT; see e.g., [33]. Another project on this approach is the recently started Synthesising Inductive Data Models project, an advanced ERC grant project of Luc de Raedt.^7^

The challenges discussed so far are challenges one could discuss equally well in a position paper with the title “Challenges Big Data Poses to Computer Science”, this raises the question: is there more to Data Science than Big Data—at least for computer scientists? To argue that there is, I end this Section with two new, related, challenges: one foundational and one conceptual.

### A foundational challenge

Many of the problems discussed so far are in essence aspects of the same problem: how do we know we have a good model? By becoming a language for other disciplines, computer science becomes *foundational* for these other sciences. This can only happen if its own foundations are secure. The selection of models from data is one of these foundations for the machine learning and data mining community.

It is well known that the so-called Problem of Induction is unsolvable [68]. That is, there is no surefire way to induce a model from a (finite) data set. The simplest way to illustrate this is by observing that there are infinitely many functions that go through finitely many given data points. Moreover, a No Free Lunch Theorems by Wolpert [70] shows that in supervised learning there is not even a best (approximating) algorithm.

This hasn’t deterred computer scientists from developing well-founded theories to learn from data. Probably the best known one is known under headings as Computational Learning Theory (COLT), Probably Approximately Correct (PAC) learning, and Statistical Learning Theory (SLT), arising out of the work of Valiant [62] and (earlier) Vapnik and Chervonenkis [65].

The approach taken in PAC learning is to search for algorithms that almost always lead to almost correct models. More specifically, following [53], PAC learning is defined as follows.

A hypothesis class $\mathcal {H}$ is agnostic PAC learnable with respect to a set *Z* and a loss function $l{:}\,Z \times \mathcal {H} \rightarrow \mathbb {R}_{+}$ if there exists a function $m_{\mathcal {H}}{:}\,(0, 1)^2 \rightarrow \mathbb {N}$ and a learning algorithm *A* with the following property:

- for every $$\epsilon , \delta \in (0, 1)$$
- for every distribution $\mathcal {D}$ over *Z*
- when running *A* on $m \ge m_{\mathcal {H}}(\epsilon , \delta )$ i.i.d. samples generated by $\mathcal {D}$
- *A* returns a hypothesis $h \in \mathcal {H}$ such that with probability at least $1 - \delta$
  $$\begin{aligned} L_{\mathcal {D}}(h) \le \min _{h' \in \mathcal {H}} L_{\mathcal {D}}(h')+ \epsilon \end{aligned}$$
  where $L_{\mathcal {D}}(h)$ is the expectation of the loss *l*(*D*, *h*) on samples *D* sampled according to $\mathcal {D}$

And then, perhaps surprisingly, there is just one property that decides whether or not a hypothesis set is PAC learnable, viz., its VC dimension. Briefly speaking, the VC dimension of $\mathcal {H}$ is the size of the largest set it can shatter, i.e., the largest set such that $\mathcal {H}$ can distinguish all its subsets—i.e., there is a hypothesis that says yes to all elements of that subset and no to all others.

Moreover, if $\mathcal {H}$ has VC dimension *d* we know that we only need a sample size of

$$\begin{aligned} O\left( \frac{d + \log (1/\delta )}{\epsilon ^2} \right) \end{aligned}$$

to achieve the $\epsilon , \delta$ bounds from the definition of PAC learning. A polynomial bound that is certainly valuable in a Big Data world. Your data may be too big to handle, but using just a sample of this size will almost always give you an almost optimal result.

In fact, PAC learning can be set up by first analyzing—with $\mathcal {H}$ finite for the two-class classification problem—how big a sample one should take. The fact that by turning these results into a general learnability definition maintain the polynomial sample size bound is a testament to the reasonableness of PAC learning.

To further illustrate this reasonableness, we briefly look at two relaxations of the PAC learning definition. Firstly, the definition requires that the sample size bound $m_{\mathcal {H}}$ holds for all hypotheses uniformly. What if we allow larger minimal sample sizes for “complex” hypotheses than for simpler ones? That relaxation leads to Structural Risk Minimization [64] in which one—conceptually, not necessarily practically—computes the optimal hypothesis by a tower of approximation, each learned the PAC learning way.

PAC learning requires that we can approximate the optimal hypothesis arbitrarily close. What if we relax this assumption? If we only require that our solution is slightly better than random guessing? The Boosting lemma [51] then tells us that we can approximate (strong) PAC learning with a weighted sum of such weak learners.

Finally, the PAC learning framework is independent of the distribution $\mathcal {D}$ the data set *D* has been sampled from. If we are willing to take $\mathcal {D}$ into account, we can get even better bounds for minimal sample sizes using Rademacher Complexity (also known as Rademacher Averages), see [53].

In other words, PAC learning is in a way as good as it gets, relaxations either approximate it or are approximated by it.

Our discussion so far was essentially for the two-class classification setting, but to a large extent it holds far more generally for supervised learning. In fact, there are also results for unsupervised learning problems such as itemset mining [49] and graph summarization [48]. These are, however, special among unsupervised problems in that they test properties. One can objectively test whether these properties hold or not, leading to a loss function that is, more or less, the same as for two-class classification. PAC learning is essentially supervised learning.

Unfortunately, not all datafied science is about predictions. With the notable exception of Hari Seldon,^8^ historians seldom attempt to predict anything. Rather, their aim is to try to understand and explain the course of history. We’ll discuss an example in some depth in the next subsection.

The point here is that many sciences are more concerned with unsupervised learning than with supervised learning. And, in general, unsupervised learning is notoriously hard to evaluate.

For example, for clustering there are various—often ad-hoc—methods to evaluate the quality of the returned clusters [27]. But there is no established way to compute how close your clustering is to the optimal clustering—in as far as the latter is even defined.

While this is not so much of a problem in a non-academic setting—or in the case where unsupervised learning is used in an exploratory data analysis phase [61] followed by a predictive analysis—it is far from ideal when the unsupervised analysis is the end product. In fact, this is exactly the reason that makes some machine learners argue [69] that unsupervised learning should never be the end product. It should only be done within a (supervised) context, giving an objective evaluation criterion: the best clustering is the one that leads to the best classifier later on.

In an extension of PAC learning, known as PAC Bayes [43], this restrictive idea has been taken up to provide objective foundations for clustering, more precisely co-clustering [52]. However, as I already stated above, in some sciences there is simply no predictive context; a historian wants to understand history, not to predict it.

However, much statistical tests and *p* values are misunderstood and misused [32], such objective measures are necessary quality control in the scientific process. It won’t take long before researchers in other datafied areas will request such objective measures for unsupervised learning.

One could argue that not even PAC learning in supervised cases gives you such measures—after all, PAC learning only tells you the results are probably as good as its gets *with the chosen hypothesis set*. However, methods like train and test or cross-validation will give you further evidence of the quality of your result. Moreover, the bootstrap will give you, empirically, all the test statistics you need [24].

All of this is lacking, in general, for the unsupervised case. There is, however, a computer science theory for unsupervised learning, viz., Algorithmic Information Theory or Kolmogorov Complexity [42]. The assumption here is that the model should be computable—not an overly stringent requirement for models one may want to do something with. More precisely the requirement is that the data set is computed by a model.

In a nutshell, fix a universal Turing machine and consider all inputs that generate our data set *D* and then halt. Out of all these inputs, choose the smallest; the length of the shortest string is known as the Kolmogorov complexity of *D*.

Why the smallest? One argument is to refer to Ockham’s razor, paraphrasing: don’t do with more for what you could do with less; see e.g., [57] for a thorough analysis of that argument.

Another argument is by compression. If the shortest input string is shorter than *D*, we have compressed *D*. Compression works by detecting regularity, hence, the shortest input string—the best possible compression—detects all regularity in *D*. Data sets for which the Kolmogorov complexity is *not* smaller than *D*,

$$\begin{aligned} K(D) \ge |D| \end{aligned}$$

are random data sets, data sets with no discernible structure.

A third, and final, argument is based on algorithmic probability [58]. Simplified, perhaps oversimplified, the reasoning is as follows. All programs computing *D* are themselves again bit strings. Kraft’s inequality [42] allows you to define a probability distribution on this set; it is slightly more complicated because the set is (countably) infinite, but this is the gist. The shortest program—the shortest bit string—computing your dataset *D* is the most likely under this distribution among all that compute *D*.

One may now wonder: why should we use this distribution? It is known as Solomonoff’s universal prior and has all the good properties one would like a non-informative prior to have. For example, it does not suffer from the re-parametrization problems most non-informative priors suffer from. See [47] for a lucid discussion of this prior, its reasonableness and the theory of universal induction it belongs to.

So, we take the shortest program, but we did not specify which universal Turing machine. The reader could wonder whether this choice makes a difference. The answer is no, because there is always a program—input string—which turns one UTM into another one; a simple consequence of being a *universal* Turing machine. Hence the complexity with regard to one UTM is at most a constant different from the complexity with regard to another one. Kolmogorov complexity analysis is always “upto a constant” for that reason.

A more serious problem is that the Kolmogorov complexity is not computable. For the simple reason that we stated the shortest program that computes *D* and then halts and it is well known that the Halting Problem is not decidable. It is upper semi-computable: dovetail over all Turing machines—input strings for your chosen UTM—and whenever one outputs *D* and then halts you have a new upper bound for the complexity of *D*. This is, however, not an effective approach in practice.

For that, note that an input string for your favorite UTM consists—often—of two parts. First a part that selects a certain Turing machine—the program—followed by a “random” part that lets that program generate *D*. In such a case, the complexity consists of two parts. Firstly, the complexity of the model (the program). Secondly, the complexity of the data given that model (the data encoded by the model).

This line of reasoning leads to the Minimum Description Length principle [34]. Like for PAC learning, we start by choosing a set of hypotheses $\mathcal {H}$. The principle can be described roughly as follows.

Given a set of models $\mathcal {H}$, the best model $H \in \mathcal {H}$ for data set *D* is the one that minimizes

$$\begin{aligned} L(H) + L(D \mid H) \end{aligned}$$

in which

- *L*(*H*) is the length, in bits, of the description of *H*
- *L*(*D*|*H*) is the length, in bits, of the description of the data when encoded with *H*.

If this looks suspiciously much like maximum likelihood with Bayes rule:

$$\begin{aligned} \mathbb {P}(H \mid D) \propto \mathbb {P}(H) \times \mathbb {P}( D \mid H) \end{aligned}$$

you are very much right. This rule was the original motivation^9^ for the inventor of MDL: Rissanen [50].

Based as it is in Algorithmic Information Theory, MDL is a sound approach to induce models from data. Indeed, it works very well for unsupervised learning, our own work on pattern set mining—starting from [54]—could be cited as an example of this. Clustering, to take that again as example, can be handled in at least two ways. Firstly, we can simply assume that each cluster $C_i$ in the data is best described by its own hypothesis $H_i$. This means that we no longer search for the single hypothesis that minimizes $L(H) + L(D \mid H)$, but for a set of hypotheses $\{ H_i \}$ and a partitioning of the data $D = \bigcup _i D_i$ minimizing the sum

$$\begin{aligned} \sum _i L(H_i) + L(D_i \mid H_i) \end{aligned}$$

Alternatively, we can employ the normalized information distance [41] based on the Kolmogorov complexity:

$$\begin{aligned} d(x, y) = \frac{\max \left\{ K(x \mid y^*), K(y \mid x^*)\right\} }{\max \{K(x), K(y)\}} \end{aligned}$$

in which $x^*$ denotes a shortest program that computes *x*.

This measure is, obviously, not computable, but it can be approximated by your favorite compression algorithm. Using that approximation to the distance, one can then use any of the traditional clustering algorithms.

The Algorithmic Information Theory approach not only works for unsupervised learning, but also for supervised learning. In fact, universal induction, which we briefly mentioned above, was developed for predictive problems [58]. Still, AIT—or, more in particular MDL—falls short of the foundational theory we are looking for.

Firstly because one has to choose a set of hypotheses, like in the PAC learning scheme, but one also has to choose an encoding scheme for MDL. In the limit, this choice may not matter. But, in practice, this choice has major impact on what one discovers—upto a constant is a problem when one has a finite data set, however big it is.

Secondly, one often has to resort to heuristic algorithms to a find a good—optimality not guaranteed—hypothesis. Moreover, it is known that MDL optimal solutions can be hard to approximate; see [2] for details. Hence, guarantees one has from the theory do not easily carry over to applications.

Finally, like PAC learning, MDL tells you which $H \in \mathcal {H}$ you should use. But unlike the supervised case, there is still no way to measure how good your choice actually is. Therefore, again unlike PAC learning, there is no way to estimate how large a sample should be to get reasonable results.

In other words, computer science has produced—at least—two beautiful theories to do well-founded induction from data. However, both theories fall short—for different reasons—if one would want to use them as inductive foundations for Data Science. For me, this is the biggest challenge computer science has to meet to become successfully the language for Data Science; even bigger than the existence of a symbolic language that one could use to reason about algorithms, the challenge we briefly discussed at the end of Sect. 2.

The fact that we have two theories that fail for different reasons, do give hope that they could somehow be combined into a universal algorithmic theory of induction. One that can be used by data scientists on whatever problem they are working on.

One way to approach to such an integration may be based on information theory. PAC Bayes—the extension we briefly discussed—is based on mutual information rather than a simple loss function. AIT is itself an information theory. Clearly, these information theories live in different universes, entropy describes the randomness at the source of messages, while AIT describes the randomness of the object (message) itself. Still, there is a strong connection between the two [35]:

$$\begin{aligned} \text {Entropy } \approx \text { expected Kolmogorov Complexity} \end{aligned}$$

A long time ago, Haussler stated [37]

It’s a dangerous thing to try and formalize an enterprise as complex and varied as machine learning...

That doesn’t mean that we shouldn’t try. Even if it wouldn’t fit all possible approaches equally well, such a theory would make computer science a far better language for data science.

### A conceptual challenge

When one collaborates with scientists from other disciplines on data science problems, most of the problems can be handled very well by our existing toolbox. Now and again, problems fit our concepts very well, but the way to approach it is not immediately obvious. One example would be outlier detection. We know very well what an outlier is [3]

An outlier is an observation which deviates so much from the other observations as to arouse suspicions that it was generated by a different mechanism

But if your colleagues asks you how to find outlying articles in a newspaper archive, the operationalization of that knowledge is not immediately clear.

Even more rarely, problems come up that do not, yet, fit the concepts we have. In this section, I discuss one such problem. Not only because it illustrates so well why data science is good for computer science: new problems are fun, but also because in this particular example, we are looking for an unsupervised solution, thus illustrating further the need for the theory I was arguing for in the previous section.

Historians now routinely have access to large digital historical archives, such as all—well, a large selection of—newspapers published in the Netherlands between 1618 and 1995 at http://www.delpher.nl/. With the advent of such resources, new kind of research questions become attainable. Before such digital archives, archive based research was limited by what human labor could achieve. Now, tools can be used to quickly select and analyze digital archives.

In one of our discussions, a colleague from the History Department of the Universiteit Utrecht suggested the following challenge. New words and concepts regularly enter our vocabulary and, especially the concepts, often lead to discussions in newspapers. For example, the concept of Darwinism entered the Dutch vocabulary not long after Darwin published his famous “On the Origin of Species” [19]. And not only our vocabulary, but also the newspapers.

At first, the articles in the newspaper mainly consisted of a discussion between proponents and opponents of the theory of evolution. But gradually this changed, Darwinism acquired a broader meaning. Rather than “just” referring to a biological theory it could also refer to a social science theory: social Darwinism [17]. A theory that upheld that humans and human society were also in that sense subject to a struggle for the survival of the fittest, “natural” selection also plays a role in the evolution of human societies.

His goal is to discover and follow such evolving (no pun intended) discourses automatically from, e.g., (digital) newspaper archives. Having access to the complete discourse and its evolution would give him a far more complete view on if, and how, and when, the concept of Darwinism became accepted by—a vast majority of—the Dutch population.

Note that this is very much an unsupervised learning task. We do not necessarily know which discourses have taken place and even less how they evolved. On first sight, this may seem like a relatively straightforward topic modeling task, more specifically a dynamic topic modeling task [10] since that technique was especially designed for that problem. However, it turned out not to be so easy.

Firstly, because it is not about the linear evolution of recognized topics such as Atomic Physics or Neuroscience, but about much more fuzzily defined topics—newspapers are not scientific journals and characteristic terms may vary between different newspapers and different epochs. Secondly, the evolution is far from linear, topics may split: the discussion goes into different directions, e.g., the discussion on biological Darwinism continues, while the discussion on social Darwinism starts. Topics may merge, they may die down and then reappear later again; for the latter, think of periodic topics like the Olympics.

This is not only a technical problem, but it is also a conceptual one. Topic modeling is very much a special case of clustering. That is, a topic is a cluster of newspaper articles together with a set of (more or less) characteristic terms. How can we decide that a cluster of one set of articles (with its characteristic terms) is similar to another set of articles (with its own characteristic terms)? Exacerbated the fact that all articles and most of the characteristic terms will be different among the two sets.

The goal of this discussion is not to solve this problem. It is to illustrate that Data Science poses new, interesting, questions to the computer science community. Moreover, it should also illustrate the need for solid foundations, both for supervised and unsupervised learning. Perhaps not with my first attempts at a solution, but certainly later, my colleague from the History department will ask: are you sure of this result? How likely is it that there is no better explanation of the course of history?

## Conclusions

In this position paper, I argued that Data Science marks the advent of datafied research in all academic research areas. Moreover, it marks the end of computer science being an area under the sole purview of computer scientists, and researchers from other areas will contribute actively to the growth of computer science’s knowledge base.

More importantly, it marks the beginning of computer science as a language for other scientists. A language that allows them to state and solve problems in their own research area that involve the storage, manipulation and/or analysis of data. This not only allows new ways to solve problems that are already well known in their research area, but also new problems—problems that were impossible to pose, let alone solve, without using computer science as a language. And this second aspect is the defining characteristic of Data Science.

This usage poses challenges to computer science. Known challenges, such as those that are already posed by Big Data: how to store, manipulate and analyze vast amounts of data of wide variety and veracity that stream in at an ever increasing rate?

But it also poses new challenges. I discussed three of these in some depth. Firstly, that our current way of writing algorithms is not directly amenable to a mathematics-style reasoning with algorithms. Secondly, and arguably the most important one, that we need a well-founded way to induce models from data—especially for unsupervised learning. If computer science is to become a language, we should have objective measures that guarantee the quality of the models we induce. And finally, conceptual challenges. Data scientists will come up with new problems for which we have no algorithms yet, including problems that do not fit in our existing data mining toolbox.

The most important message of this position paper is, however, that the advent of Data Science is good for computer science. Not only will we be presented with many new challenges, the solutions we design become more and more important to an ever increasing community of data scientists. It is truly an exciting time to be a computer scientist.

## Footnotes

1 I hope that the datafication of all sciences will do the same for the—more famous—two cultures Snow [56] distinguished, by giving them a common language.

2 This also happens when computer scientists publish in not-primarily computer science journals, see e.g., [39] for an example.

3 In fact it formed the basis of Eddington’s famous first conformation of general relativity, the deflection being twice as large as predicted by Newton’s laws.

4 Which we will briefly discuss in Sect. 3.1.

5 Also available online as the NIST Digital Library of Mathematical Functions at dmlf.nist.gov

6 https://www.openml.org/.

7 http://synth.cs.kuleuven.be/.

8 The inventor of psychohistory, an algorithmic science that allows him to predict the future in probabilistic terms. A fictional character in Isaac Asimov’s Foundation series [5].

9 As it was for the universal induction we mentioned above.
